# Porphyrin photosensitizers in photodynamic therapy and its applications

**DOI:** 10.18632/oncotarget.20189

**Published:** 2017-08-11

**Authors:** Jiayuan Kou, Dou Dou, Liming Yang

**Affiliations:** ^1^ Department of Pathophysiology, Harbin Medical University, Harbin, PR China; ^2^ Department of Biochemistry and Molecular Biology, Harbin Medical University, Harbin, PR China; ^3^ Department of Physiology and Pathophysiology, School of Basic Medical Sciences, Peking University Health Science Center, Beijing, PR China

**Keywords:** porphyrin photosensitizers, photodynamic therapy, tumor, application

## Abstract

In 1841, the extraction of hematoporphyrin from dried blood by removing iron marked the birth of the photosensitizer. The last twenty years has witnessed extensive research in the application of photodynamic therapy (PDT) in tumor-bearing (or other diseases) animal models and patients. The period has seen development of photosensitizers from the first to the third generation, and their evolution from simple to more complex entities. This review focuses on porphyrin photosensitizers and their effect on tumors, mediated via several pathways involved in cell necrosis, apoptosis or autophagic cell death, and the preventive and therapeutic application of PDT against atherosclerosis.

## INTRODUCTION

**Figure 1:**
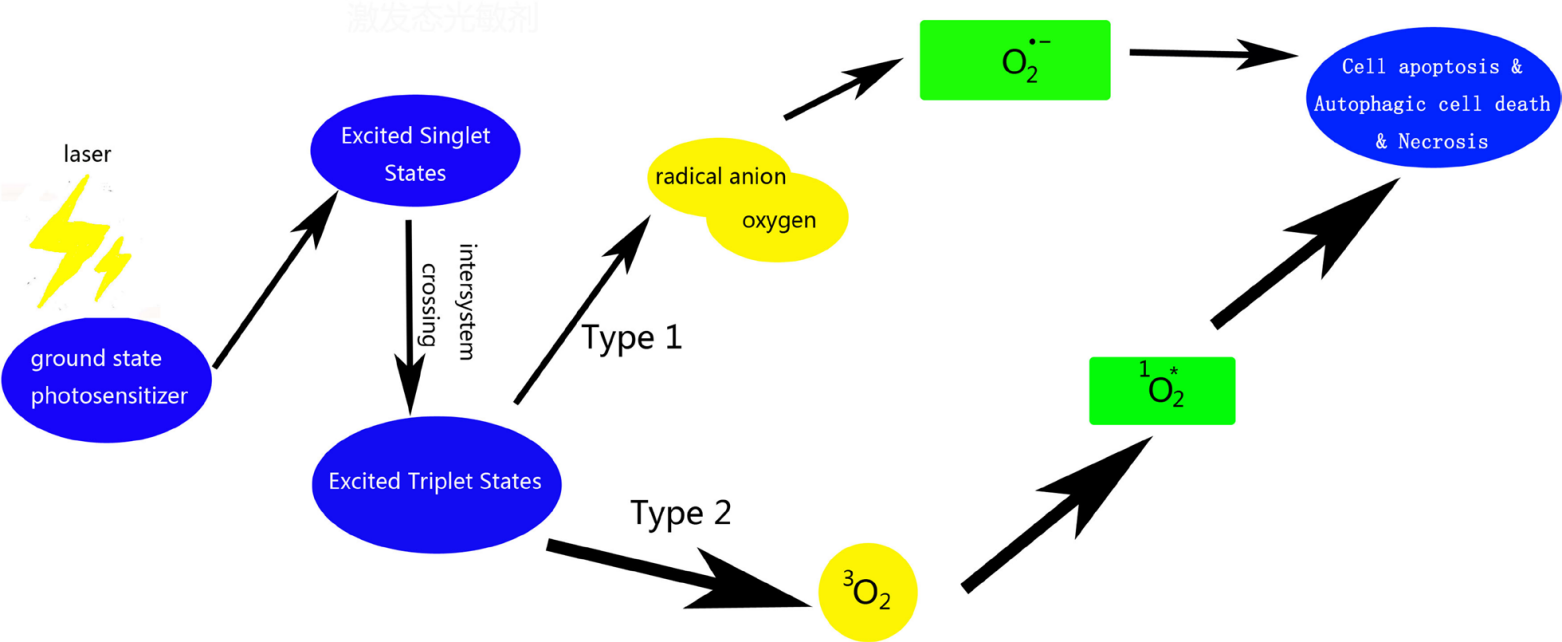
Scheme of photosensitizer activating and ROS producing involved in PDT

Photodynamic therapy (PDT) employs a combination of photosensitizer, light, and molecular oxygen, to selectively target cells like tumor cells *via* cytotoxic activity [1]. Tumor and macrophage cells have a preferential uptake of photosensitizers. These photosensitizers are activated on exposure to light and become photosensitizers’ triplet, which, react with molecular oxygen to produce reactive oxygen species (ROS) [2]. The hydroxyl radical is another reason which leads to the reaction between the photosensitizer and molecular oxygen, including the Fenton reaction of hydrogen peroxide, which in turn produces more hydroxyl radicals [3]. These cytotoxic molecules induce a series of biological reactions that ultimately lead to cell death [4] (Figure 1). The outcomes of PDT depend on the nature of the cells, as well as the on the properties and localization of photosensitizer and the illumination conditions [5]. Its obvious advantage is that cause negligible damage to the surrounding normal tissues and has little systemic effects. Moreover, there is no obvious mechanism of acquiring resistance to PDT, which makes it a promising modality for treatment of skin, esophageal, and lung cancers, as well as other non-neoplastic diseases such as atherosclerosis, macular degeneration, and rheumatoid arthritis [2, 6]. In the last century, two Nobel prizes were awarded in the field of PDT (Table 1). Extensive research has been carried out in basic and clinical area using PDT; however, the potential application of PDT against atherosclerosis and tumors has not seen much development. This review summarizes the available research evidence on the use of porphyrin photosensitizers and the application of PDT against tumors and atherosclerotic lesions. The objective is to provide a better understanding of PDT for new comers to the field.

**Table 1:** Brief history of PDT

Year	Individuals	Events
1841	Scherer	Discovery of hematoporphyrin by removing iron from dried blood
1861–1871	L. Pasteur and P. Bert	Discovery of phototoxicity
1867	J.L.W. Thudichum	Fluorescence spectrum of this red substance (hematoporphyrin) as well as fluorescence.
1871	F.Hoppe-Seyler	Naming of red substance (hematoporphyrin).
1874	Schultz	Description of a porphyria patient (errors in heme biosynthesis).
1895–1903	N.R.Finsen	Phototherapy (Nobel prize in 1903)
1897–1904	O.Raaband H. von Tappeiner	First reports on phototherapy
1904	H. von Tappeiner	Introduction of the term “photodynamic action”
1903–1905	—	First “before-and-after” photographs of patients (eosin+light)
1908–1913	W.Hausmann, F.Meyer-Betz	Many PDT experiments with hematoporphyrin on paramecia, erythrocytes, mice, guinea pigs, and humans.
1924	—	A.Policard saw red porphyrin fluorescence in tumors and first observation from tumors
1925	H.Fischer	Examination of porphyrins (Nobel prize in 1929).
1945	S.Scwarz	Radiosensitization with porphyrins.
1959	D.Harman	Proposed the free radical theory of ageing and disease.
1960–1967	R.Lipson E.Baldes	Synthesis of HpD.
1970	H.Kautsky G.Herzberg	Active oxygen.
1975	Z.Malik M.Djaldetti	ALA for PpIX induction.
1983–1993	T.J.Dougherty, *et al.*	Photofrin^®^.
1990	J.Kennedy R.Pottier	Clinical application of ALA.

### The development of the porphyrin photosensitizers

PDT has three functional elements: photosensitizer, optical wavelength of light and molecular oxygen [6]. The fundamental biological reaction in PDT involves absorption of light energy by the photosensitizers and its subsequent transfer to induce chemical alteration [7–9].

The first generation photosensitizers, hematoporphyrin derivative (HpD) and photofrin II (a purified form of HPD) were employed in early clinical trials of PDT (Table 2) [10]. HPD was shown to be effective in brain, laryngeal, lung, skin, gastric, and esophageal carcinomas to a certain degree [11–13]. In fact, hematoporphyrin, but not its derivatives, was discovered during the early period. *In vitro* studies of photosensitizer uptake in brain tumor samples showed significantly higher mean HpD uptake in glioblastoma multiforme as compared to that in anaplastic astrocytoma [11]. Cerebral glioma patients treated with adjuvant PDT following surgical resection were associated with better prognosis [14]. While it was not effective for tumor-localization after purification, HpD appeared which added acetic-sulfuric acid mixtures based on the origin structure.

**Table 2:** First generation photosensitizers and their targets

Photosensitizer	Wavelength	Targets	Authors	Year
Hematoporphyrin derivative (HpD)	630 nm	100 patients with malignant mesothelioma	Clarke CP, Knight SR, Daniel FJ, *et al.* [89].	2006
		Patients with high grade glioma	Stylli SS, Kaye AH, MacGregor L, *et al.* [90].	2005
		Brain tumor tissue sample	Stylli SS, Howes M, MacGregor L, *et al.* [11].	2004
		142 patients with advanced gastrointestinal cancers	Jin ML, Yang BQ, Zhang W, *et al.* [91].	1992
		Patients with subfoveal choroidal neovascularization	Schmidt-Erfurth U, Miller J, Sickenberg M, *et al.*[92].	1990
Photofrin	630 nm	Mice bearing radiation-induced fibrosarcoma tumors	Qiu H, Kim MM, Penjweini R,*et al.* [93].	2017
		23 patients with advanced colorectal cancer	Sun BO, Li W, Liu N, *et al.* [15].	2016
	635 nm	4T1 BALB/c female mice (Breast cancer)	Wang, X., Hu, J., Wang, P., *et al.* [94].	2015
	625 nm	Male Wistar rats (Oral cancer/dysplasia)	Nauta, J.M., van Leengoed, H.L., Witjes, M.J., *et al.*[95].	1997
	635 nm	OVCAR3 Nude mice (Ovarian)	Peterson, C.M., Reed, R., Jolles, C.J., *et al.*[96].	1992

However, after a large number of clinical studies, the limitations of photofrin, such as its complex composition and low light absorption rate were identified. In one pre-clinical study, the photodynamic therapy dose, apparent reacted singlet oxygen, and predict local control rate were measured for photofrin-mediated PDT of radiation-induced fibrosarcoma tumors *via* mice-bearing models [15]. In addition, photofrin-mediated PDT treatment of young patients with advanced colorectal cancer showed amelioration of clinical symptoms and reduction in the incidence of complications [16]. However, due to the relatively short wavelength of light, only a small amount of light can enter into the tumor through the skin, while most of the light is blocked on the skin surface; this essentially results in cutaneous photosensitive toxicity [17, 18]. These disadvantages promoted the development of second-generation photosensitizers.

Compared with the first generation photosensitizer, the composition and structure of the second generation photosensitizer are clear, and the photosensitivity, absorption spectrum and tissue selectivity have been greatly improved. To a certain extent, the first generation of photosensitizer has complex components, which is very bad for the selectivity of tissue and the stability of photodynamic damage intensity. Most of the second generation photosensitizers are based on porphyrin structure, such as benzoporphyrins, purpurins, texaphyrins, phthalocyanines, naphthalocyanines, and protoporphyrin IX (PpIX). PpIX was shown to have has a longer wavelength absorption in erythroleukemia cells [4, 8, 18, 19]. It is a precursor of heme, and is involved in the metabolism of heme through the combination of mitochondrial transport proteins. Another commonly used photosensitizer is 5-aminolevulinic acid (ALA), the biological precursor of PpIX [18]. A phase I trial of ALA-mediated PDT in 11 patients with for oral leukoplakia demonstrated the benefits and the safe dose of ALA-PDT could be administered with a low light dose of up to 4J/cm^2^ [20]. Other photosensitizers, like mono-aspartyl chlorin e6 (NPe6), temoporfin, and hexylpyropheophorbide (HPPH), are based on the chlorin structure (Table 3) [6].

**Table 3:** The second generation photosensitizers and their targets

Photosensitizer	Wavelength	Targets	Authors	Year
Benzoporphyrin derivative monoacid ring A (BPD-MA), vertoporfin	689 nm	Subjects with non-facial PWS	Tournas JA, Lai J, Truitt A et al. [33].	2009
		Tumor tissue in a mouse tumor model	Richter AM, Waterfield E, Jain AK et al. [97].	1993
Meso-tetrakis (4-sulfonatophenyl) porphyrin (TPPS)		Osteosarcoma cells	Duchi S, Sotgiu G, Lucarelli E et al. [98].	2013
N-aspartyl chlorin e6, NPe6	660 nm	7 patients with bile duct carcinoma	Nanashima A, Abo T, Nonaka T, et al.[99].	2012
Aminolevulinic acid (5-ALA)	635 nm	9 patients with deep-seated contrast enhancing brain tumors	Rapp M, Kamp M, Steiger HJ, et al. [100].	2014
		Patients with suspected malignant gliomas	Díez Valle R, Slof J, Galván J, et al.[101].	2014
Temoporfin or m-THPC (Foscan(^®)^)	652 nm	Rat model employing a radioactive lipid label and (14)C-temoporfin.	Decker C, Schubert H, May S, et al. [102].	2013
TSPP	—	Wistar male rats bearing 256 Walker carcinosarcoma	Clichici S, Filip A, Daicoviciu D, et al. [28].	2010
HPPH		Mice and rat tumor models	Spernyak J A, White III W H, Ethirajan M, et al. [103].	2010

Other second-generation photosensitizers were designed to meet specific demands, such as the new mitochondria-targeting photosensitizers, DLC (delocalized lipophilic cations) which can preferentially be localized in mitochondria. Based on DLC, three DLCs-porphyrin conjugates: a core modified porphyrin-rhodamine B cation, a core modified porphyrin-mono-triphenyl phosphonium cation, and a core modified porphyrin-di-tPP cation, were prepared [21]. The chemical structure of the original photosensitizer was modified to improve the problem of organelle targeting and to increase the anti-tumor effect of drugs. After di-imide reduction, disulfonated tetraphenyl porphine [TPPS(2a)] was transferred into disulfonated tetraphenyl chlorin [TPCS(2a)] for better induced activation of gelonin, which delayed tumor growth in athymic mice on subcutaneous irradiation [22]. There is a possibility that most new porphyrins are excited at a higher wavelength illumination; therefore, a deeper light penetration of photosensitizer is needed for further studies [23].

Physicochemical interactions of TPCS(2a) and TPPS(2a) have been widely studied to determine their properties such as solubilization and aggregation in aqueous media [24–26]. and the researchers could provide the data in an extensive survey when a clinical trial is on the plan. Production of ROS is important for the therapeutic effect of PDT, and the singlet oxygen is considered as the most important ROS [8]. So, the ability of porphyrins to generate singlet oxygen is a key element of enquiry in PDT-related studies. Electrochemical sensors have been used for continuous real-time monitoring of the effect of photosensitizer-induced PDT reactions on the functional integrity of the bacterial cell envelope [27]. The effect of PDT with TSPP (meso-tetrakis (4-sulfonatophenyl) porphine) on the production levels of ROS and the metalloproteinase 2 activities has evoked much interest, as has the relationship between the local accumulation of photosensitizers and the intratumor histological alterations [28]. Fluorescent probes have been used to directly monitor the formation of singlet oxygen and hydroxyl radicals during photodynamic therapy [29]. Safety is a key concern, including identification of the minimum energy levels of light and concentrations. Phototoxicity of two porphyrin photosensitizers, TPPS4 and MgTPPS4, was investigated *in vitro* on Hela cells to determine the illumination parameters that were associated with eradication of HIV-1 infectivity without damaging the infected leukocytes [30, 31]. The influence of these electrical charges on the iontophoretic delivery of photosensitizers was further evaluated *in vitro* and *in vivo*, in an attempt to achieve maximum accumulation of photosensitizers, whilst ensuring minimum retention in skin tissues [32].

As time caught up with those ideas, many investigators were not satisfied with outcomes of research on single drug. A combination of PDT and pulsed dye laser (PDL) was assessed in a proof-of-concept preliminary clinical trial [33]. The combinations of two or three photosensitizers were proposed to be more effective. Weyergang *et al*. evaluated PDT as neoadjuvant to epidermal growth factor receptor (EGFR) targeting drugs, Cetuximab, Erlotinib, and Tyrphostin AG1478. The results showed that these three drugs in combination with PDT showed a superior anti-tumor effect by causing prolonged inhibition of extracellular signal–regulated kinase (ERK). In addition, poor response of cells on EGFR activation deficiency was overcome by the combination of PDT and Gefitinib, as elucidated by Postiglione I *et al.* [34, 35].

### The bridge between 2nd and 3rd generation

Chemical modifications for more accurate targeting have led to the discovery of the next generation photosensitizers, for example mTHPC, introduced by Berenbaum [36]. It is questionable whether the drug is the second or the third generation photosensitizer [37]. Based on these characteristics, considerable efforts have been devoted to develop specific carriers for delivery of photosensitizers in order to avoid phototoxicity to normal tissues, such as skin [38].

### The third generation

Third-generation photosensitizers are now being developed to improve the PDT outcomes (Table 4). Currently the two main loci of research are gene engineering mediated PDT and use of nanotechnology in PDT. In a study, photosensitizers were injected post transfection of neoplastic cells by firefly luciferase, which could activate the photosensitizer in the organism, leading to the destruction of neoplastic cells [39].

**Table 4:** Third generation photosensitizers and their targets

Photosensitizer	Wavelength	Targets	Authors	Year
Gold-NanoclusteredHyaluronan Nano-Assemblies		Orthotopic breast tumor model	Han HS, Choi KY, Lee H, et al. [104].	2016
Chlorin E6 (Ce6)+Upconversion nanoparticles	980 nm, 405 nm	THP-1 macrophages	Xing Zhu, Hao Wang, Longbin Zheng, et al. [46].	2015
Photofrin+ gap junctional intercellular communication (Connexin 32)	—	Transfected HeLa cells and in the xenograft tumors	Wu D, Fan L, Xu C, et al. [105].	2015
Ce6+tumor-targeting nanogel	—	Tumor-bearing mice experiments	Kim JY, Choi WI, Kim M, et al. [64].	2013
Ce6+ChitoUDCA nanoparticles	200–400 nm	HuCC-T1 human cholangiocarcinoma cells	Lee HM, Jeong YI, Kim do H, et al. [41].	2013
ICG-loaded nanospheres coated with chitosan	800–805 nm	Infectious pathogens	Nagahara A, Mitani A, Fukuda M, et al. [43].	2013

Chlorin E6 (Ce6), one of photosensitizers, was incorporated into nanoparticles through the formation of ion complexes to enhance absorption by the tumor and to improve the levels of ROS generation [40]. Ce6 was also developed to improve cancer imaging and treatment due to the strong NIR (in the near-infrared range of 650–800 nm) absorption and the capability of encapsulating in the gold vesicles (GVs) [41]. There is no doubt that such behavior is not limited to tumor. Indocyanine green (ICG)-loaded nanospheres were designed by Nagahara *et al.* to improve the bactericidal effect of PDT on *Porphyromonas gingivalis* [42]. In order to improve antimicrobial effects and to reduce damage to peripheral tissues, the absorption of photosensitizers by microbial cells should also be enhanced [43]. Optimized photophysical characteristics such as the generation of cytotoxic ROS and the depth of light penetration are also important; hence our group loaded Ce6 onto upconversion nanoparticles to afford greater penetration than that achieved with Ce6 alone [44]. Recently, a new kind of green titania was facilely synthesized, which showed much enhanced near NIR absorption [45]. This feature enables it to be stimulated with 980 nm Laser in the combined PDT and photothermal therapy (PTT), which is greatly beneficial for improving tissue penetration depth.

### Mechanism of PDT application to tumor

For the treatment of tumor, surgery is not a radical treatment for some kinds or extent of cancer. Radiation therapy and chemotherapy are not effective enough and have several side effects. Therefore new approaches for treatment of cancer are necessary. There are three distinct mechanisms involved; one of these is direct phototoxicity to tumor cells, leading to apoptosis, necrosis or autophagic cell death (Figure 1). The other two are destruction of the tumor vascular system and immune-mediated inflammatory damage to tumor cells (Table 5).

**Table 5:** Photosensitizers used in tumor cells and the potential acting molecular pathways

Photosensitizer	Targets	Mechanism	Subtype of tumor	Authors	Year
Hypericin	MCF-7 as well as in MDA-MB-231 cells	Activation of caspase 3/7 and apoptosis	Human breast adenocarcinoma	Kimáková P, Solár P, Fecková B, et al. [109].	2017
Photofrin	Human ESCC cellline SHEEC and parental normal cellline SHEE, primary culture cells	Controlling for vascular factors	Esophageal cancer	Gao S, Liang S, Ding K, et al. [106].	2016
Photofrin	ASTC-a-1 cells	Bcl-2-interacting mediator of cell death	Lung adenocarcinoma	Wang X, He X, Hu S, et al. [107].	2015
HMME	Human tongue squamous cell carcinoma Tca8113 cells *in vitro*	Activation of caspase-3 and apoptosis	Human tongue squamous carcinoma	Lai X, Ning F, Xia X, et al. [108].	2015
5- ALA	Human urothelial cancer cells and human umbilical vein endothelial cells, *in vivo* PDT with a tumor-bearing animal model	The ALA-PDT decreased levels of mitochondrial membrane potential and induced cell death mainly via apoptosis in these cells.	Human urothelial cancer	Inoue K, Fukuhara H, Kurabayashi A, et al. [56].	2013
HPPH	16 adult patients (median age, 65 years) with biopsy-proved primary or recurrent resectable head and neck squamous cell carcinoma	—	Head and neck squamous cell carcinoma	Rigual NR, Shafirstein G, Frustino J, et al. [59].	2013

Direct phototoxic effect of PDT on tumor cells involves irreversible photo damage to specific targets, such as membranes and organelles, at the molecular level. Other cell death pathways are usually considered as useful targets to induce, and thus increase photokilling in tumor cells harboring defects in apoptotic pathways, which is a crucial step in carcinogenesis and therapy resistance [46]. Focusing on the molecular differences of cell death mechanisms induced by PDT will certainly provide valuable clues for the development of new therapeutic modalities and drug selectivity to improve the efficacy of PDT against cancer cells.

### Apoptosis

Apoptosis is characterized by nuclear condensation and general cellular shrinkage, and involves a series of caspases, endonucleases, and other enzymes [47]. During the first study of PDT-mediated activation of apoptosis, little was known regarding the mechanisms involved in apoptosis. However, it was clear that initiation of the process could be triggered by the translocation of cytochrome c from mitochondria to the cytosol [48, 49]. The basic method to analyze the effect of killing cells is always related to some original data, for example, the increase in ratio of apoptotic cells with increase in light dosage or intracellular photosensitizers concentration. However, those skills are not enough, with the development of the exploration, and more sophisticated substance related to apoptosis needs to be emphasized.

The apoptotic caspases are involved in two converging pathways: extrinsic and intrinsic. When an apoptotic signal is released, all caspases can be activated as the initiator caspase or an upstream caspase [50]. Preliminary studies by Kessel *et al.* indicated that apoptosis inhibition resulted from translocation of photosensitizers from the membrane to the cytosol during irradiation, which was associated with photo damage to caspase-3, a major substance during induction of apoptosis, leading to selective photo damage to procaspases-9, and -3 [51]. Pretreatment with specific caspase-6 inhibitor abolished the PDT-induced cleavage of lamin A/C and subsequent apoptosis, which suggests that the cleavage of lamin A/C is enhanced by activation of caspase-6, and that it is crucial for apoptotic induction [52].

The initiation of apoptosis was shown to be inhibited by over-expression of Bcl-2 [47]. The Bcl-2 protein family includes at least 20 members, and until now, the role of Bcl-2 in PDT is not clear [53]. Studies have shown that over-expression of Bcl-2 in cells inhibited PDT-induced apoptosis to a certain extent; however, in another study, the levels of Bcl-2 protein increased following an increase of efficiency of PDT [18, 54]. Kim *et al.* detected the effects of Bcl-2 over-expression with aluminum phthalocyanines as the photosensitizing agent in PDT. The results showed that caspase-3 activation was accompanied by the enhanced mitochondrial cytochrome c release under 50 mJ/cm^2^ light dose of PDT treatment, and a stronger apoptosis reaction [47, 55]. However, if Bcl-2 over-expression leads to stabilization of Bax, selective Bcl-2 photo damage can result in a high Bax: Bcl-2 ratio and an enhanced apoptotic response to mitochondrial photo damage.

### Autophagic cell death

Autophagic cell death is characterized by double-membrane autophagic vacuoles, also called autophagosomes [46]. Some studies have shown that PDT-induced cell death is closely related to autophagy activation [56–58]. Buytaert *et al.* reported that PDT with hypericin *via* endoplasmic reticulum (ER) pathway led to an immediate loss of SERCA2 protein levels, causing disruption of Ca2+ homeostasis and cell death. And, it was causal to cell killing. At that time, Bax/Bak gateway was repaired to prevent apoptosis, but to undergo autophagy-associated cell death as revealed by electron microscopy and biochemical analysis [56].

### Necrosis

In general, the photosensitizers that targeting the mitochondria and endoplasmic reticulum, can promote cell apoptosis by inducing oxidative stress within a certain range,; however, localization of the photosensitizer in the cell membrane or the lysosome probably pushes cells to necrosis due to blockade of apoptotic pathway [59]. In certain PDT-induced necrosis, some photosensitizers directly tend to induce cell necrosis, rather than apoptosis-induced secondary necrosis [46].

In recent years, therapeutic effects of ALA-based PDT against urothelial carcinoma were shown to be enhanced by deferoxamine, a kind of traditional iron chelating agents, while PDT-induced cell damage to the surrounding tissues was found to be under the safe threshold [60]. Coincidentally, Li *et al.* reported improved therapeutic effects of PDT by combining bortezomib with verteporfin-based PDT. The results showed stronger activation of apoptosis in endothelial cells and greater suppression of tumor growth with combination therapy, as compared to that with individual treatments [61]. Gold nanorods were used as a photothermal therapy agent in combination with Ce6-based PDT, and the whole complex system was found to target the tumor site more efficiently [62].

Minimizing the photoxicity of PDT is an important aspect of application of PDT against tumors. One study showed that the expected and unexpected effects observed were pain, and inflammatory reactions after PDT for skin cancer [63]. The pain intensity was correlated with the anatomical localization of the lesion. The patients reported a higher intensity of pain in lesions located on the head and neck as compared to those on the trunk and limbs. Some researchers intended to change to a new photoactive drug in order to reduce the phototoxic effect on the peritumoral normal tissues. For example, Rigual *et al.* used surgery and HPPH-based PDT in patients with head and neck squamous cell carcinoma [64]. Others have favored lowering the dose of PDT without reducing the photo killing of tumor cells, with or without the concomitant use of anticancer drugs. For example, Ahn *et al* reported that a combination of PDT and anticancer drug cisplatin was more effective in reducing tumor growth in mice xenograft [65]. In addition, treatment of tumor cells with sub-lethal PDT induced the formation of angiogenic factors and survival molecules, and this self-protective reaction made tumor cells resistant to treatment. Elsewhere, a combination of anti-inflammatory drug celecoxib and PDT was shown to strengthen the original apoptotic response and anti-tumor efficiency induced by PDT alone [66].

### Mechanism PDT application to cardiovascular disease

Over the past decades, appreciation of the role of PDT on cardiovascular system, especially atherosclerosis, has burgeoned because PDT can not only act on tumor cells, but also other unwanted cells (Table 6). Atherosclerosis (also known as arteriosclerotic vascular disease), has long been considered as a lipid deposition disease accompanied by an ongoing inflammatory response. The macrophages and smooth muscle cells phagocytic oxidized low-density lipoprotein until the ability of cholesterol efflux from the lipid-loaded cells is damaged [67, 68].

**Table 6:** The mechanisms of photosensitizers-mediated PDT in the cardiovascular-related studies

Photosensitizer	Targets	Mechanism	Author	Year
Ce6	THP-1 macrophages	Apoptosis	Xing Zhu, Hao Wang, Longbin Zheng, et al. [41].	2015
L-SR15	murine macrophage Raw 264.7 cells	Preferential destruction of pro-inflammatory macrophages in atheromata might attenuate plaque growth or rupture-prone vulnerability	Lee DK, Choi Y, Shon SM, et al. [1].	2011
5-ALA	rabbit postballoon injury model for ALA-photoangioplasty	Mitochondria, cytosolic membrane	Kwon OC, Yoon HJ, Kim KH, et al. [71].	2008
chlorin e6	30 specimens of human aorta and 15 specimens of human coronary arteries	Lysosomes, endosomes	Biały D, Derkacz A, Wawrzyńska M, et al. [81].	2003
HPD	Forty Japanese White rabbits	Golgi apparatus, plasma membrane	Usui M, Asahara T, Naitoh Y, et al. [69].	1999
Photofrin	rabbits	Golgi apparatus, plasmamembrane	Amemiya T, Nakajima H, Katoh T, et al. [68].	1999
Photofrin	Twelve Yucatan miniswine	Golgi apparatus, plasma membrane	Hsiang YN, Crespo MT, Machan LS, Bower RD, Todd ME [67].	1994

Atherosclerotic plaques mainly include stable and vulnerable plaques [69]. Stable atherosclerotic plaques, usually asymptomatic, contain extracellular matrix and smooth muscle cells. While vulnerable plaques are composed of foam cells, macrophages, and the extracellular matrix, which is usually weak and prone to rupture [70]. Exposure of substances such as collagen to circulation following plaque rupture initiates the formation of thrombus in the lumen [71]. In order to avoid the occurrence of acute cardiovascular events, new therapeutic strategies are needed to improve treatment efficacy in atherosclerosis and to make the vulnerable plaque more stable, or reduce the intracellular content of plaque, or induce the effective outflow of lipid.

Hsiang *et al.* determined the feasibility of treating atherosclerotic stenoses with photodynamic therapy. Although the results demonstrated resolution in stenoses in some miniswines, questions concerning light dosimetry, mechanism of action, and long-term effects remain to be determined [72]. Amemiya *et al.* performed photodynamic therapy using the photosensitizer, photofrin, and the results showed widening of vascular lumen with reduction in intima and media, which suggests that PDT effectively reduced atherosclerotic lesions [73]. HpD-based PDT was used to treat intimal hyperplasia in rabbits by Usui *et al.* The results showed decreased smooth muscle cell growth and suppressed intimal hyperplasia response [74]. In 2001, Yamaguchi *et al.* observed that Lu-Tex-based PDT reduced atherosclerotic lesions of experimental graft coronary artery disease, which contributed to treat accelerated atherosclerosis associated with transplantation of new ideas [75]. More and more research results indicated that people should go further, not content with “whether” but properties in detail. In 2003, Kereiakes DJ *et al.* assessed the safety and tolerability of Motexafin lutetium-mediated phototherapy in patients undergoing percutaneous coronary intervention with stent deployment [76]. The results showed that there are rare serious dose-limiting toxicities and side effects (paresthesia and rash). In 2008, Kwon *et al.* reported that ALA-based PDT significantly reduced the atheromatous plaque without causing damage to the medial wall, although the smooth muscle cells persisted in the aortic media. In the future, further optimization of PDT is needed to eliminate the residual smooth muscle cells in order to prevent restenosis [77].

Focusing on the mechanism was needed to apply PDT on atherosclerosis clinically better on the basis of some research. Macrophages play an important role in atherogenesis by releasing cytokines and taking up modified low-density lipoprotein, resulting in the accumulation of lipids within plaque and damage to cholesterol efflux and the formation of a necrotic lipid core. With further release of proteolytic enzymes, macrophages are more likely to promote plaque rupture [78]. Every element in the process may potentially be modulated by PDT. Macrophages whose membrane contains scavenger receptor, can be recognized by scavenger receptor-based PDT and targeted [79, 80]. Moreover, oxidized-LDL also can be used as a delivery vehicle for photosensitizers to the macrophages, enhancing the targeting and therapeutic effects of PDT in the treatment of atherosclerosis [81].

In addition to the macrophages, smooth muscle cells also play an essential role in atherogenesis, and several studies are focusing on the effects of PDT on such cells. The photosensitizer PpIX of the dosage and the illumination energy was optimized to the appropriate lower range, and it can be sure that the main way of cell death is apoptosis the main cell death pathway could be the most important way to ensure that the cell death was apoptosis [82]. Tian *et al.* also measured the apoptotic or necrotic ratio of smooth muscle cells induced with PpIX-based PDT. The results showed that the cellular viability reduced with higher illumination energy and higher intracellular PpIX dosage [83]. Waksman *et al.* determined the PDT induced reduction of plaque inflammation and repopulation in smooth muscle cell-rich plaque models [78]. In addition to the two kinds of cells mentioned above, the proliferation and the invasive migration of fibroblasts, endothelial cells, and matrix protein cross-links repair were also reported to be enhanced following PDT [84–86]. The reconstituted endothelium had a beneficial effect on preventing the influx of macrophages into the intimal layer, but the precise mechanisms are not clear. Some studies showed that PDT enhanced vessel healing and repair [78]. However, PDT-induced arterial wall weakening and aneurismal dilation have also been reported, which suggests the need for further research using more desirable illumination energy or photosensitizer dosage, or even new photosensitizers [87]. The relation between the photosensitizer and its targets was regarded to be based on the covalent conjugation of a photosensitizer and cell-surface receptors [88]. Therefore, this process may need to be investigated further in detail.

Based on the prior experience with tumors, diagnostic application of PDT has been suggested in the context of cardiovascular disease. PDT may discriminate the normal and calcified segments of atherosclerotic plaques in real-time imaging; it may also be possible for PDT to estimate the various forms and stages of atherosclerosis in the future [87].

## CONCLUSIONS AND PERSPECTIVES

PDT represents a multidisciplinary diagnostic and therapeutic modality with potential application in a variety of disciplines, and its future application development space is only limited by the imagination of researchers [6]. This review provides a summary of current knowledge base on the application of PDT for treatment of tumors and atherosclerosis, rather than identifying the entire spectrum of the potential use of PDT. Furthermore, the clinical use of PDT in high-risk surgical procedures may need to be given serious consideration for different individual conditions, tumors, and atherosclerosis locations. For new and better photosensitizers, some characteristics have been generally accepted as criteria for ideal photosensitizers (Table 7). There is now a general consensus on the lack of any obvious damage caused by preventive PDT. However, there is a controversy of how much PDT can possibly be used in prevention, not in the treatment. We placed a special emphasis on decreasing phototoxicity. The photosensitizer will be more precise on location and prevention of direct damage by PDT to the surrounding tissues. It will be better to have memory ability with the photosensitizer and it will follow the order of the debris of atherosclerotic plaques to reach the targets.

**Table 7:** Criteria for ideal photosensitizers

Characteristics
Chemically pure and specific composition.
Stability at room temperature.
Minimal dark toxicity.
Only be cytotoxic in the presence of light at defined wavelength.
Preferential retention by target tissues.
Excellent photochemical reactivity with high triplet state yields and long triplet state life times.
Be inexpensive and commercially available.
Be easy to dissolve in the body's tissue fluids.

## References

[1] Lee DK, Choi Y, Shon SM, Schellingerhout D, Park JE, Kim DE (2011). Atorvastatin and clopidogrel interfere with photosensitization in vitro. Photochem Photobiol Sci.

[2] Ochsner M (1997). Photophysical and photobiological processes in the photodynamic therapy of tumours. J Photochem Photobiol B.

[3] St Denis TG, Dai T, Izikson L, Astrakas C, Anderson RR, Hamblin MR, Tegos GP (2011). All you need is light: antimicrobial photoinactivation as an evolving and emerging discovery strategy against infectious disease. Virulence.

[4] Josefsen LB, Boyle RW (2008). Photodynamic therapy: novel third-generation photosensitizers one step closer?. Br J Pharmacol.

[5] Plonka J, Latocha M (2012). Photodynamic therapy in the treatment of breast cancer. Pol Merkur Lekarski.

[6] Agostinis P, Berg K, Cengel KA, Foster TH, Girotti AW, Gollnick SO, Hahn SM, Hamblin MR, Juzeniene A, Kessel D, Korbelik M, Moan J, Mroz P (2011). Photodynamic therapy of cancer: an update. CA Cancer J Clin.

[7] Huang YY, Tanaka M, Vecchio D, Garcia-Diaz M, Chang J, Morimoto Y, Hamblin MR (2012). Photodynamic therapy induces an immune response against a bacterial pathogen. Expert Rev Clin Immunol.

[8] Berg K, Selbo PK, Weyergang A, Dietze A, Prasmickaite L, Bonsted A, Engesaeter BO, Angell-Petersen E, Warloe T, Frandsen N, Hogset A (2005). Porphyrin-related photosensitizers for cancer imaging and therapeutic applications. J Microsc.

[9] Allison RR, Sibata CH (2010). Oncologic photodynamic therapy photosensitizers: a clinical review. Photodiagnosis Photodyn Ther.

[10] Spikes JD (1990). Chlorins as photosensitizers in biology and medicine. J Photochem Photobiol.

[11] Stylli SS, Howes M, MacGregor L, Rajendra P, Kaye AH (2004). Photodynamic therapy of brain tumours: evaluation of porphyrin uptake versus clinical outcome. J Clin Neurosci.

[12] Yoshida T, Saeki T, Ohashi S, Okudaira T, Lee M, Yoshida H, Maruoka H, Ito H, Funasaka S, Kato H (1995). Clinical study of photodynamic therapy for laryngeal cancer. Nihon Jibiinkoka Gakkai Kaiho.

[13] Chissov VI, Sokolov VV, Filonenko EV, Menenkov VD, Zharkova NN, Kozlov DN, Polivanov Iu N, Prokhorov AM, Pykhov RL, Smirnov VV (1995). Clinical fluorescent diagnosis of tumors using photosensitizer photogem. Khirurgiia.

[14] Favilla I, Favilla ML, Gosbell AD, Barry WR, Ellims P, Hill JS, Byrne JR (1995). Photodynamic therapy: a 5-year study of its effectiveness in the treatment of posterior uveal melanoma, and evaluation of haematoporphyrin uptake and photocytotoxicity of melanoma cells in tissue culture. Melanoma Res.

[15] Sun BO, Li W, Liu N (2016). Curative effect of the recent photofrin photodynamic adjuvant treatment on young patients with advanced colorectal cancer. Oncol Lett.

[16] Toratani S, Tani R, Kanda T, Koizumi K, Yoshioka Y, Okamoto T (2016). Photodynamic therapy using Photofrin and excimer dye laser treatment for superficial oral squamous cell carcinomas with long-term follow up. Photodiagnosis Photodyn Ther.

[17] Gomer CJ (1991). Preclinical examination of first and second generation photosensitizers used in photodynamic therapy. Photochem Photobiol.

[18] Oleinick NL, Morris RL, Belichenko I (2002). The role of apoptosis in response to photodynamic therapy: what, where, why, and how. Photochem Photobiol Sci.

[19] Malik Z, Lugaci H (1987). Destruction of erythroleukaemic cells by photoactivation of endogenous porphyrins. Br J Cancer.

[20] Decker C, Schubert H, May S, Fahr A (2013). Pharmacokinetics of temoporfin-loaded liposome formulations: correlation of liposome and temoporfin blood concentration. J Control Release.

[21] Rajaputra P, Nkepang G, Watley R, You Y (2013). Synthesis and in vitro biological evaluation of lipophilic cation conjugated photosensitizers for targeting mitochondria. Bioorg Med Chem.

[22] Berg K, Nordstrand S, Selbo PK, Tran DT, Angell-Petersen E, Hogset A (2011). Disulfonated tetraphenyl chlorin (TPCS2a), a novel photosensitizer developed for clinical utilization of photochemical internalization. Photochem Photobiol Sci.

[23] Klyta M, Ostasiewicz P, Jurczyszyn K, Dus K, Latos-Grazynski L, Pacholska-Dudziak E, Ziolkowski P (2011). Vacata- and divacataporphyrin: new photosensitizers for application in photodynamic therapy-an in vitro study. Lasers Surg Med.

[24] Nardo L, Kristensen S, Tonnesen HH, Hogset A, Lilletvedt M (2012). Solubilization of the photosensitizers TPCS(2a) and TPPS(2a) in aqueous media evaluated by time-resolved fluorescence analysis. Pharmazie.

[25] Lilletvedt M, Tonnesen HH, Hogset A, Sande SA, Kristensen S (2011). Evaluation of physicochemical properties and aggregation of the photosensitizers TPCS2a and TPPS2a in aqueous media. Pharmazie.

[26] Lilletvedt M, Tonnesen HH, Hogset A, Nardo L, Kristensen S (2010). Physicochemical characterization of the photosensitizers TPCS2a and TPPS2a 1. Spectroscopic evaluation of drug--solvent interactions. Pharmazie.

[27] Komagoe K, Kato H, Inoue T, Katsu T (2011). Continuous real-time monitoring of cationic porphyrin-induced photodynamic inactivation of bacterial membrane functions using electrochemical sensors. Photochem Photobiol Sci.

[28] Clichici S, Filip A, Daicoviciu D, Ion RM, Mocan T, Tatomir C, Rogojan L, Olteanu D, Muresan A (2010). The dynamics of reactive oxygen species in photodynamic therapy with tetra sulfophenyl-porphyrin. Acta Physiol Hung.

[29] Price M, Reiners JJ, Santiago AM, Kessel D (2009). Monitoring singlet oxygen and hydroxyl radical formation with fluorescent probes during photodynamic therapy. Photochem Photobiol.

[30] Bernstein ZP, Dougherty T, Gollnick S, Schwartz SA, Mahajan SD, Kepner J, Sumlin A, Stewart C, Wallace P, Adal A, Walder H, Poiesz B (2008). Photopheresis in HIV-1 infected patients utilizing benzoporphyrin derivative (BPD) verteporfin and light. Curr HIV Res.

[31] Binder S, Kolarova H, Tomankova K, Bajgar R, Daskova A, Mosinger J (2011). Phototoxic effect of TPPS4 and MgTPPS4 on DNA fragmentation of HeLa cells. Toxicol In Vitro.

[32] Gelfuso GM, Gratieri T, Souza JG, Thomazine JA, Lopez RF (2011). The influence of positive or negative charges in the passive and iontophoretic skin penetration of porphyrins used in photodynamic therapy. Eur J Pharm Biopharm.

[33] Tournas JA, Lai J, Truitt A, Huang YC, Osann KE, Choi B, Kelly KM (2009). Combined benzoporphyrin derivative monoacid ring photodynamic therapy and pulsed dye laser for port wine stain birthmarks. Photodiagnosis Photodyn Ther.

[34] Postiglione I, Chiaviello A, Aloj SM, Palumbo G (2013). 5-aminolaevulinic acid/photo-dynamic therapy and gefitinib in non-small cell lung cancer cell lines: a potential strategy to improve gefitinib therapeutic efficacy. Cell Prolif.

[35] Weyergang A, Selbo PK, Berg K (2013). Sustained ERK inhibition by EGFR targeting therapies is a predictive factor for synergistic cytotoxicity with PDT as neoadjuvant therapy. Biochim Biophys Acta.

[36] Rassmussen-Taxdal DS, Ward GE, Figge FH (1955). Fluorescence of human lymphatic and cancer tissues following high doses of intravenous hematoporphyrin. Cancer.

[37] Senge MO (2012). mTHPC--a drug on its way from second to third generation photosensitizer?. Photodiagnosis Photodyn Ther.

[38] Nishiyama N, Morimoto Y, Jang WD, Kataoka K (2009). Design and development of dendrimer photosensitizer-incorporated polymeric micelles for enhanced photodynamic therapy. Adv Drug Deliv Rev.

[39] Babincova M, Sourivong P, Babinec P (2000). Gene transfer-mediated intracellular photodynamic therapy. Med Hypotheses.

[40] Lee HM, Jeong YI, Kim DH, Kwak TW, Chung CW, Kim CH, Kang DH (2013). Ursodeoxycholic acid-conjugated chitosan for photodynamic treatment of HuCC-T1 human cholangiocarcinoma cells. Int J Pharm.

[41] Lin J, Wang S, Huang P, Wang Z, Chen S, Niu G, Li W, He J, Cui D, Lu G, Chen X, Nie Z (2013). Photosensitizer-loaded gold vesicles with strong plasmonic coupling effect for imaging-guided photothermal/photodynamic therapy. ACS nano.

[42] Nagahara A, Mitani A, Fukuda M, Yamamoto H, Tahara K, Morita I, Ting CC, Watanabe T, Fujimura T, Osawa K, Sato S, Takahashi S, Iwamura Y (2013). Antimicrobial photodynamic therapy using a diode laser with a potential new photosensitizer, indocyanine green-loaded nanospheres, may be effective for the clearance of Porphyromonas gingivalis. J Periodontal Res.

[43] Kushibiki T, Hirasawa T, Okawa S, Ishihara M (2013). Responses of cancer cells induced by photodynamic therapy. J Healthc Eng.

[44] Zhu X, Wang H, Zheng L, Zhong Z, Li X, Zhao J, Kou J, Jiang Y, Zheng X, Liu Z, Li H, Cao W, Tian Y (2015). Upconversion nanoparticle-mediated photodynamic therapy induces THP-1 macrophage apoptosis via ROS bursts and activation of the mitochondrial caspase pathway. Int J Nanomedicine.

[45] Mou J, Lin T, Huang F, Shi J, Chen H (2017). A New Green Titania with Enhanced NIR Absorption for Mitochondria-Targeted Cancer Therapy. Theranostics.

[46] Buytaert E, Dewaele M, Agostinis P (2007). Molecular effectors of multiple cell death pathways initiated by photodynamic therapy. Biochim Biophys Acta.

[47] Kim HR, Luo Y, Li G, Kessel D (1999). Enhanced apoptotic response to photodynamic therapy after bcl-2 transfection. Cancer Res.

[48] Agarwal ML, Clay ME, Harvey EJ, Evans HH, Antunez AR, Oleinick NL (1991). Photodynamic therapy induces rapid cell death by apoptosis in L5178Y mouse lymphoma cells. Cancer Res.

[49] Li P, Nijhawan D, Budihardjo I, Srinivasula SM, Ahmad M, Alnemri ES, Wang X (1997). Cytochrome c and dATP-dependent formation of Apaf-1/caspase-9 complex initiates an apoptotic protease cascade. Cell.

[50] Boatright KM, Salvesen GS (2003). Mechanisms of caspase activation. Curr Opin Cell Biol.

[51] Kessel D (2002). Relocalization of cationic porphyrins during photodynamic therapy. Photochem Photobiol Sci.

[52] Shahzidi S, Brech A, Sioud M, Li X, Suo Z, Nesland JM, Peng Q (2013). Lamin A/C cleavage by caspase-6 activation is crucial for apoptotic induction by photodynamic therapy with hexaminolevulinate in human B-cell lymphoma cells. Cancer Lett.

[53] Hanahan D, Weinberg RA (2000). The hallmarks of cancer. Cell.

[54] Almeida RD, Manadas BJ, Carvalho AP, Duarte CB (2004). Intracellular signaling mechanisms in photodynamic therapy. Biochim Biophys Acta.

[55] He J, Agarwal ML, Larkin HE, Friedman LR, Xue LY, Oleinick NL (1996). The induction of partial resistance to photodynamic therapy by the protooncogene BCL-2. Photochem Photobiol.

[56] Buytaert E, Callewaert G, Hendrickx N, Scorrano L, Hartmann D, Missiaen L, Vandenheede JR, Heirman I, Grooten J, Agostinis P (2006). Role of endoplasmic reticulum depletion and multidomain proapoptotic BAX, BAK proteins in shaping cell death after hypericin-mediated photodynamic therapy. FASEB J.

[57] Kessel D, Vicente MG, Reiners JJ (2006). Initiation of apoptosis and autophagy by photodynamic therapy. Lasers Surg Med.

[58] Schroder M, Kaufman RJ (2005). ER stress and the unfolded protein response. Mutat Res.

[59] Kessel D, Luo Y, Deng Y, Chang CK (1997). The role of subcellular localization in initiation of apoptosis by photodynamic therapy. Photochem Photobiol.

[60] Inoue K, Fukuhara H, Kurabayashi A, Furihata M, Tsuda M, Nagakawa K, Fujita H, Utsumi K, Shuin T (2013). Photodynamic therapy involves an antiangiogenic mechanism and is enhanced by ferrochelatase inhibitor in urothelial carcinoma. Cancer Sci.

[61] Li Z, Agharkar P, Chen B (2013). Therapeutic enhancement of vascular-targeted photodynamic therapy by inhibiting proteasomal function. Cancer Lett.

[62] Kim JY, Choi WI, Kim M, Tae G (2013). Tumor-targeting nanogel that can function independently for both photodynamic and photothermal therapy and its synergy from the procedure of PDT followed by PTT. J Control Release.

[63] Blanco KC, Inada NM, Silva AP, Stringasci MD, Buzza HH, Ramirez DP, Salvio AG, Moriyama LT, Kurachi C, Bagnato VS (2017). A Multicenter Clinical Study of Expected and Unexpected Side Reactions During and After Skin Cancer Treatment by Photodynamic Therapy. Skinmed.

[64] Rigual NR, Shafirstein G, Frustino J, Seshadri M, Cooper M, Wilding G, Sullivan MA, Henderson B (2013). Adjuvant intraoperative photodynamic therapy in head and neck cancer. JAMA Otolaryngol Head Neck Surg.

[65] Ahn JC, Biswas R, Mondal A, Lee YK, Chung PS (2014). Cisplatin enhances the efficacy of 5-aminolevulinic acid mediated photodynamic therapy in human head and neck squamous cell carcinoma. Gen Physiol Biophys.

[66] Song J, Chen Q, Xing D (2013). Enhanced apoptotic effects by downregulating Mcl-1: evidence for the improvement of photodynamic therapy with Celecoxib. Exp Cell Res.

[67] Libby P, Ridker PM, Maseri A (2002). Inflammation and atherosclerosis. Circulation.

[68] Yin Y, Pastrana JL, Li X, Huang X, Mallilankaraman K, Choi ET, Madesh M, Wang H, Yang XF (2013). Inflammasomes: sensors of metabolic stresses for vascular inflammation. Front Biosci.

[69] Ross R (1999). Atherosclerosis--an inflammatory disease. N Engl J Med.

[70] Finn AV, Nakano M, Narula J, Kolodgie FD, Virmani R (2010). Concept of vulnerable/unstable plaque. Arterioscler Thromb Vasc Biol.

[71] Didangelos A, Simper D, Monaco C, Mayr M (2009). Proteomics of acute coronary syndromes. Curr Atheroscler Rep.

[72] Hsiang YN, Crespo MT, Machan LS, Bower RD, Todd ME (1994). Photodynamic therapy for atherosclerotic stenoses in Yucatan miniswine. Can J Surg.

[73] Amemiya T, Nakajima H, Katoh T, Rakue H, Miyagi M, Ibukiyama C (1999). Photodynamic therapy of atherosclerosis using YAG-OPO laser and Porfimer sodium, and comparison with using argon-dye laser. Jpn Circ J.

[74] Usui M, Asahara T, Naitoh Y, Katoh T, Ibukiyama C (1999). Photodynamic therapy for the prevention of intimal hyperplasia in balloon-injured rabbit arteries. Jpn Circ J.

[75] Yamaguchi A, Woodburn KW, Hayase M, Hoyt G, Robbins RC (2001). Photodynamic therapy with motexafin lutetium (Lu-Tex) reduces experimental graft coronary artery disease. Transplantation.

[76] Kereiakes DJ, Szyniszewski AM, Wahr D, Herrmann HC, Simon DI, Rogers C, Kramer P, Shear W, Yeung AC, Shunk KA, Chou TM, Popma J, Fitzgerald P (2003). Phase I drug and light dose-escalation trial of motexafin lutetium and far red light activation (phototherapy) in subjects with coronary artery disease undergoing percutaneous coronary intervention and stent deployment: procedural and long-term results. Circulation.

[77] Kwon OC, Yoon HJ, Kim KH, Kim HT, Yoon YH, Kim JK (2008). Fluorescence kinetics of protoporphyrin-IX induced from 5-ALA compounds in rabbit postballoon injury model for ALA-photoangioplasty. Photochem Photobiol.

[78] Waksman R, McEwan PE, Moore TI, Pakala R, Kolodgie FD, Hellinga DG, Seabron RC, Rychnovsky SJ, Vasek J, Scott RW, Virmani R (2008). PhotoPoint photodynamic therapy promotes stabilization of atherosclerotic plaques and inhibits plaque progression. J Am Coll Cardiol.

[79] Hamblin MR, Miller JL, Ortel B (2000). Scavenger-receptor targeted photodynamic therapy. Photochem Photobiol.

[80] Brasseur N, Langlois R, La Madeleine C, Ouellet R, van Lier JE (1999). Receptor-mediated targeting of phthalocyanines to macrophages via covalent coupling to native or maleylated bovine serum albumin. Photochem Photobiol.

[81] de Vries HE, Moor AC, Dubbelman TM, van Berkel TJ, Kuiper J (1999). Oxidized low-density lipoprotein as a delivery system for photosensitizers: implications for photodynamic therapy of atherosclerosis. J Pharmacol Exp Ther.

[82] Star WM (1997). Light dosimetry in vivo. Phys Med Biol.

[83] Li Q, Cheng J, Peng C, Li Z, Shi S, Liang H, Tian Y, Zhang Z, Cao W (2010). Apoptosis of vascular smooth muscle cells induced by photodynamic therapy with protoporphyrin IX. Biochem Biophys Res Commun.

[84] Heckenkamp J, Aleksic M, Gawenda M, Breuer S, Brabender J, Mahdavi A, Aydin F, Brunkwall JS (2004). Modulation of human adventitial fibroblast function by photodynamic therapy of collagen matrix. Eur J Vasc Endovasc Surg.

[85] Overhaus M, Heckenkamp J, Kossodo S, Leszczynski D, LaMuraglia GM (2000). Photodynamic therapy generates a matrix barrier to invasive vascular cell migration. Circ Res.

[86] Waterman PR, Overhaus M, Heckenkamp J, Nigri GR, Fungaloi PF, Landis ME, Kossodo SC, LaMuraglia GM (2002). Mechanisms of reduced human vascular cell migration after photodynamic therapy. Photochem Photobiol.

[87] Bialy D, Derkacz A, Wawrzynska M, Bednarkiewicz A, Ziolkowski P, Nowosad H, Strek W (2003). *In vitro* photodynamic diagnosis of atherosclerotic wall changes with the use of mono-l-aspartyl chlorin e6. A preliminary report. Kardiol Pol.

[88] Gabeler EE, van Hillegersberg R, Sluiter W, Kliffen M, Statius van Eps RG, Honkoop J, Carlier SG, van Urk H (2003). Arterial wall strength after endovascular photodynamic therapy. Lasers Surg Med.

[89] Clarke CP, Knight SR, Daniel FJ, Seevanayagam S (2006). Management of malignant mesothelioma by decortication and adjunct phototherapy. Asian Cardiovasc Thorac Ann.

[90] Stylli SS, Kaye AH, MacGregor L, Howes M, Rajendra P (2005). Photodynamic therapy of high grade glioma - long term survival. J Clin Neurosci.

[91] Jin ML, Yang BQ, Zhang W, Ren P (1990). Review of photodynamic therapy for gastrointestinal tumours in the past 6 years in China. J Photochem Photobiol B.

[92] Schmidt-Erfurth U, Miller J, Sickenberg M, Bunse A, Laqua H, Gragoudas E, Zografos L, Birngruber R, van den Bergh H, Strong A, Manjuris U, Fsadni M, Lane AM (1998). Photodynamic therapy of subfoveal choroidal neovascularization: clinical and angiographic examples. Graefes Arch Clin Exp Ophthalmol.

[93] Qiu H, Kim MM, Penjweini R, Finlay JC, Busch TM, Wang T, Guo W, Cengel KA, Simone CB, Glatstein E, Zhu TC (2017). A Comparison of Dose Metrics to Predict Local Tumor Control for Photofrin-mediated Photodynamic Therapy. Photochem Photobiol.

[94] Wang X, Hu J, Wang P, Zhang S, Liu Y, Xiong W, Liu Q (2015). Analysis of the in vivo and in vitro effects of photodynamic therapy on breast cancer by using a sensitizer, sinoporphyrin sodium. Theranostics.

[95] Nauta JM, van Leengoed HL, Witjes MJ, Nikkels PG, Star WM, Vermey A, Roodenburg JL (1997). Photofrin-mediated photodynamic therapy of chemically-induced premalignant lesions and squamous cell carcinoma of the palatal mucosa in rats. Int J Oral Maxillofac Surg.

[96] Peterson CM, Reed R, Jolles CJ, Jones KP, Straight RC, Poulson AM (1992). Photodynamic therapy of human ovarian epithelial carcinoma, OVCAR-3, heterotransplanted in the nude mouse. Am J Obstet Gynecol.

[97] Duchi S, Sotgiu G, Lucarelli E, Ballestri M, Dozza B, Santi S, Guerrini A, Dambruoso P, Giannini S, Donati D, Ferroni C, Varchi G (2013). Mesenchymal stem cells as delivery vehicle of porphyrin loaded nanoparticles: effective photoinduced in vitro killing of osteosarcoma. J Control Release.

[98] Price M, Heilbrun L, Kessel D (2013). Effects of the oxygenation level on formation of different reactive oxygen species during photodynamic therapy. Photochem Photobiol.

[99] Rapp M, Kamp M, Steiger HJ, Sabel M (2014). Endoscopic-assisted visualization of 5-aminolevulinic acid-induced fluorescence in malignant glioma surgery: a technical note. World Neurosurg.

[100] Diez Valle R, Slof J, Galvan J, Arza C, Romariz C, Vidal C, researchers Vs (2014). Observational, retrospective study of the effectiveness of 5-aminolevulinic acid in malignant glioma surgery in Spain (The VISIONA study). Neurologia.

[101] Wong SJ, Campbell B, Massey B, Lynch DP, Cohen EE, Blair E, Selle R, Shklovskaya J, Jovanovic BD, Skripkauskas S, Dew A, Kulesza P, Parimi V (2013). A phase I trial of aminolevulinic acid-photodynamic therapy for treatment of oral leukoplakia. Oral Oncol.

[102] Petri A, Yova D, Alexandratou E, Kyriazi M, Rallis M (2012). Comparative characterization of the cellular uptake and photodynamic efficiency of Foscan(R) and Fospeg in a human prostate cancer cell line. Photodiagnosis Photodyn Ther.

[103] Rizzi M, Tonello S, Estevao BM, Gianotti E, Marchese L, Reno F (2016). Verteporfin based silica nanoparticle for in vitro selective inhibition of human highly invasive melanoma cell proliferation. J Photochem Photobiol B.

[104] Maiolino S, Moret F, Conte C, Fraix A, Tirino P, Ungaro F, Sortino S, Reddi E, Quaglia F (2015). Hyaluronan-decorated polymer nanoparticles targeting the CD44 receptor for the combined photo/chemo-therapy of cancer. Nanoscale.

[105] Lamch L, Bazylinska U, Kulbacka J, Pietkiewicz J, Biezunska-Kusiak K, Wilk KA (2014). Polymeric micelles for enhanced Photofrin II (R) delivery, cytotoxicity and pro-apoptotic activity in human breast and ovarian cancer cells. Photodiagnosis Photodyn Ther.

[106] Gao S, Liang S, Ding K, Qu Z, Wang Y, Feng X (2016). Specific cellular accumulation of photofrin-II in EC cells promotes photodynamic treatment efficacy in esophageal cancer. Photodiagnosis Photodyn Ther.

[107] Wang X, He X, Hu S, Sun A, Lu C (2015). Involvement of Bim in Photofrin-mediated photodynamically induced apoptosis. Cell Physiol Biochem.

[108] Lai X, Ning F, Xia X, Wang D, Tang L, Hu J, Wu J, Liu J, Li X (2015). HMME combined with green light-emitting diode irradiation results in efficient apoptosis on human tongue squamous cell carcinoma. Lasers Med Sci.

[109] Kimáková P, Solár P, Fecková B, Sačková V, Solárová Z, Ilkovičová L, Kello M (2017). Photoactivated hypericin increases the expression of SOD-2 and makes MCF-7 cells resistant to photodynamic therapy. Biomed Pharmacother.

